# RNA-Seq-Based Whole Transcriptome Analysis of IPEC-J2 Cells During Swine Acute Diarrhea Syndrome Coronavirus Infection

**DOI:** 10.3389/fvets.2020.00492

**Published:** 2020-08-13

**Authors:** Fanfan Zhang, Weifeng Yuan, Zhiquan Li, Yuhan Zhang, Yu Ye, Kai Li, Zhen Ding, Yunyan Chen, Ting Cheng, Qiong Wu, Yuxin Tang, Deping Song

**Affiliations:** ^1^Key Laboratory for Animal Health of Jiangxi Province, Jiangxi Agricultural University, Nanchang, China; ^2^Department of Preventive Veterinary Medicine, College of Animal Science and Technology, Jiangxi Agricultural University, Nanchang, China; ^3^Institute of Animal Husbandry and Veterinary Medicine, Jiangxi Academy of Agricultural Sciences, Nanchang, China; ^4^College of Animal Science and Technology, Jiangxi Agricultural University, Nanchang, China

**Keywords:** SADS-CoV, RNA-Seq, transcriptome, gene expression, porcine serum amyloid A-3

## Abstract

The new emergence of swine acute diarrhea syndrome coronavirus (SADS-CoV) has resulted in high mortality in suckling pigs in China. To date, the transcriptional expression of host cells during SADS-CoV infection has not been documented. In this study, by means of RNA-Seq technology, we investigated the whole genomic expression profiles of intestinal porcine epithelial cells (IPEC-J2) infected with a SADS-CoV strain SADS-CoV-CH-FJWT-2018. A total of 24,676 genes were identified: 23,677 were known genes, and 999 were novel genes. A total of 1,897 differentially expressed genes (DEGs) were identified between SADS-CoV-infected and uninfected cells at 6, 24, and 48 h post infection (hpi). Of these, 1,260 genes were upregulated and 637 downregulated. A Gene Ontology enrichment analysis revealed that DEGs in samples from 6, 24, and 48 hpi were enriched in 79, 383, and 233 GO terms, respectively, which were mainly involved in immune system process, response to stimulus, signal transduction, and cytokine–cytokine receptor interactions. The 1,897 DEGs were mapped to 109 KEGG Ontology (KO) pathways classified into four main categories. Most of the DEGs annotated in the KEGG pathways were related to the immune system, infectious viral disease, and signal transduction. The mRNA of porcine serum amyloid A-3 protein (SAA3), an acute phase response protein, was significantly upregulated during the infection. Over-expressed SAA3 in IPEC-J2 cells drastically inhibited the replication of SADS-CoV, while under-expressed SAA3 promoted virus replication. To our knowledge, this is the first report on the profiles of gene expression of IPEC-J2 cells infected by SADS-CoV by means of RNA-*Seq* technology. Our results indicate that SADS-CoV infection significantly modified the host cell gene expression patterns, and the host cells responded in highly specific manners, including immune response, signal and cytokine transduction, and antiviral response. The findings provide important insights into the transcriptome of IPEC-J2 in SADS-CoV infection.

## Introduction

Coronavirus (CoV) is an enveloped single-stranded positive-sense RNA virus in the family *Coronaviridae*, subfamily *Coronavirinae*, which includes four genera, *Alphacoronavirus* (α-CoV)*, Betacoronavirus* (β-CoV), *Gammacoronavirus* (γ-CoV), and *Deltacoronavirus* (δ-CoV). Coronavirus may infect a variety of mammalian species and birds, resulting in gastroenteritis, encephalitis, and respiratory symptoms (1). So far, six pathogenic CoVs have been identified in pigs, including four α-CoVs [porcine epidemic diarrhea virus (PEDV), transmissible gastroenteritis virus (TGEV), porcine respiratory coronavirus (PRCV), and the newly emerged Swine acute diarrhea syndrome coronavirus (SADS-CoV)], one β-CoV [porcine hemagglutinating encephalomyelitis virus (PHEV)], and one δ-CoV [porcine deltacoronavirus (PDCoV)] (2–4). SADS-CoV, also named as swine enteric alphacoronavirus (SeACoV) and porcine enteric alphacoronavirus (PEAV), is a newly emerged pathogenic coronavirus in pigs. SADS-CoV is a highly pathogenic enteric CoV that was firstly discovered in a fatal diarrhea outbreak in Guangdong province, China, in January 2017, leading to the death of 24,693 newborn piglets (3, 5, 6). In 2018, we identified and isolated a field strain of SADS-CoV, designated as SADS-COV-CH-FJWT-2018, in Fujian, a neighboring province of Guangdong, in China (7). In February 2019, severe diarrhea outbreak caused by SADS-CoV re-emerged in suckling piglets within 7 days of birth in Southern China (8). A retrospective investigation indicated that SADS-CoV emerged in China at least as early as August 2016, and a high prevalence rate (43.53%) was found in diarrheal samples tested (4). The manifestations of the illness in neonatal piglets induced by SADS-CoV were characterized by vomiting, severe diarrhea, and nearly 100% mortality (3, 6). Clinical lesions indicated that SADS-CoV mainly infected the small intestine of piglets, especially the jejunum and ileum, and could cause severe atrophic enteritis of 1-week-old piglets, resulting in a high morbidity, and mortality (8, 9). Currently, there are no commercial vaccines and antiviral agents for SADS-CoV, and the pathogenesis is roughly unknown as well.

Previous pathogenesis studies on coronaviruses have revealed that there are extensive and complex interactions between viruses and hosts, which is of import in terms of disease prevention and control caused by these pathogens. Host cell responses to virus infection involve complex interactions between cellular and viral networks (10–12). Most viruses attempt to change host cellular processes and organism systems to improve the efficiency of virus infection, and, on the other hand, the cells employ multiple mechanisms in responses to generating an antiviral state (13, 14). As reported, CoV infection can cause alterations in the transcription and translation patterns, cell cycle, cytoskeleton, and apoptosis pathways of host cells (15–22). With traditional approaches, it is difficult to explore the intricate and mass interactions between viruses and hosts/cells. Next-generation' sequencing (NGS) technology has provided a powerful tool for the studies of emerging and re-emerging human and animal pathogens/diseases. NGS technology, including whole genome sequencing, RNA-Seq, and single cell sequencing, has been extensively applied in genome sequencing, gene expression profiling analysis, and pathogen detection (15, 23–25).

Up to date, the study on mechanisms of infection and pathogenesis of SADS-CoV is limited. A study reported that SADS-CoV infection could antagonize interferon production and lead to immune evasion (26). However, the underneath mechanisms remain roughly unknown. There is no report on the transcriptome profile of intestinal epithelial cells during SADS-CoV infection. Thus, to address the global gene expressions profile of intestinal epithelial cells in SADS-CoV infection, a porcine intestinal epithelial cell line of IPEC-J2 was used as a model. The whole transcriptomics of the cells at 6, 24, and 48 h post infection (hpi) of SADS-CoV was determined via RNA-Seq technology. Subsequently, differentially expressed genes (DEGs) were screened, and the gene functions, signaling pathways associated with viral infection, and pathogenesis were analyzed. This study would increase our knowledge on the transcriptomics landscape of SADS-CoV infected small intestinal cells and shed light on future explorations of the mechanisms of SADS-CoV infection.

## Materials and Methods

### Cells and Virus

IPEC-J2 porcine epithelial cells were propagated in Dulbecco's modified eagle medium (DMEM) (Gibco, USA) supplemented with 10% fetal bovine serum (FBS), 1% penicillin/streptomycin (Gibco), 1% insulin-transferrin-sodium selenite (Roche), and 5 ng/ml of human epidermal growth factor (Invitrogen). The Vero (ATCC CCL-81) cells were maintained in minimal essential medium (MEM) (Gibco) supplemented with 10% heat-inactivated fetal bovine serum, 5% L-glutamine, 100 U/ml of penicillin G, and 100 μg/ml streptomycin at 37°C in a humidified 5% CO_2_ incubator (27). The SADS-CoV CH/FJWT/2018 (passage 10) virus was plaque purified for three times in Vero 81 cells supplemented with a final concentration of 10 μg/ml trypsin in DMEM as described in our previous study (28).

### Growth Kinetics of SADS-CoV in IPEC-J2 Cells

To determine the growth kinetics of the isolated SADS-CoV strain SADS-COV-CH-FJWT-2018, confluent IPEC-J2 cells in six-well plates were washed twice with D-Hanks and then inoculated with 500 μl of viral supernatants containing SADS-COV-CH-FJWT-2018 (P10) at an MOI of 1. After 2 h of incubation at 37°C with 5% CO_2_, the six-well plates were washed with D-Hanks to remove unabsorbed viruses and added 2 ml of DMEM containing 10 μg/ml trypsin. Afterwards, the six-well plates were incubated at 37°C with 5% CO_2_, and cell culture supernatants were then collected at different time intervals (0, 6, 12, 18, and up to 72 hpi). The virus titer for each time point in each sample collected was determined by 50% tissue culture infective dose (TCID_50_) (29) and an SYBR Green real-time RT-PCR assay previously established in our laboratory. Samples harvested at each time point were independently repeated three times, and the mean value, and standard deviation (SD) were calculated.

### RNA Extraction, cDNA Library Construction, and *RNA-Seq*

For RNA-Seq, nine samples from IPEC-J2 cells infected with SADS-CoV at 6, 24, and 48 hpi with three samples from each time-point (group named as A_6h, A_24h, and A_48h, respectively), and nine mock-infected negative controls with corresponding time points (group named as B_6h, B_24h, and B_48h, respectively), were collected. Firstly, the cell culture supernatants were removed, and the cells were washed twice with 0.01 M sterile phosphate-buffered saline (pH 7.2). The cells were then lysed with 1 ml Trizol Reagent (TaKaRa, Japan) for RNA extraction according to the manufacturer's instructions. The concentrations of extracted RNA were determined by using a NanoDrop 2,000 spectrophotometer (Thermo Scientific, USA), and the integrity of purified RNA was evaluated via a RNA integrity number (RIN) by an Agilent Bioanalyzer 2,100 system (Agilent Technologies, USA). Sequencing libraries were generated using NEBNext UltraTM RNA Library Prep Kit for Illumina (NEB, USA) following manufacturer's protocols. Briefly, mRNA was firstly purified from total RNA using poly-T oligo-attached magnetic beads, and then digested into short fragments by adding fragmentation reagents. Subsequently, first strand cDNA was synthesized using random N6 primer and M-MLV Reverse Transcriptase. Second strand cDNA synthesis was subsequently performed using DNA Polymerase I and RNase H. Remaining overhangs were converted into blunt ends via exonuclease/polymerase activities. After adenylation of 3′ ends of DNA fragments, adaptors with a hairpin loop structure were ligated to prepare for hybridization (NEBNest Adaptor Kit). Then, cDNA fragments were separated by agarose gel electrophoresis, and the fragments of 250–300 bp in length were selected and purified with AMPure XP system (Beckman Coulter, USA). Then, a 3 μl measurement of USER Enzyme (NEB, USA) was incubated with size-selected, adaptor-ligated cDNA at 37°C for 15 min followed by 5 min at 95°C before PCR. Then PCR was performed to generate a cDNA library with Phusion High-Fidelity DNA polymerase, universal PCR primers, and Index (X) Primers (NEB lab, USA). Finally, PCR products were purified (AMPure XP system), and library quality was assessed on the Agilent Bioanalyzer 2,100 system. The libraries were sequenced on Illumina HiSeq 2,500 platform using the paired-end technology by Novogene Co., Ltd (Beijing, China).

### Bioinformatic Analysis of *RNA-Seq* Data

The raw reads were qualified by removing reads containing adapter, poly-N and low-quality. The plus reads were clean reads, and all of the clean reads were then separated according to the barcodes. The adapter and barcode sequences were trimmed. The trimmed clean reads were assembled and mapped to the pig genome (WASHUC2.69) in Ensemble using Hisat2 (v2.0.5), and the fragments per kilobase million (FPKM) of each gene were then calculated based on the length of the gene and read counts mapped to genes. A negative binomial distribution-based model in DESeq2 was used to determine the differential expressed genes (DEGs) among the treatments. The fold change of genes between SADS-CoV infected and mock-infected samples were calculated under log_2_|FoldChange| (log_2_|FC|) ≥ 1 and a false discovery rate (FDR) adjusted *p* (*p*_adj_) < 0.05 based on a method described previously (30). The numbers of novel and known genes were statistically calculated. To predict the major biological and molecular functions of these DGEs, Gene Ontology (GO) enrichment analysis (http://www.geneontology.org) was carried out and visualized by the cluster Profiler R package (31). All of the DEGs generated were functionally analyzed against KEGG (32). The significance of all GO and KEGG terms was corrected by controlling the *p*_adj_-value.

### Verification of Differential Expressed Genes by Real-Time qRT-PCR

To validate the accuracy of the RNA-Seq results, 20 biologically related DEGs (IFIT1, IFIT2, IL-6, etc.) and a housekeeping gene beta actin were randomly selected for real-time qRT-PCR validation (primer information is listed in Additional file 1: Table S1). The relative expression values were normalized, with the beta actin gene serving as an internal control. After amplification, the relative fold change of the differentially expressed genes was calculated through the 2^−ΔΔCT^ algorithm.

### The Effects of Serum Amyloid A3 Protein (SAA3) on SADS-CoV Replication

A recombinant IPEC-J2-OE-SAA3 cell line, an over-expressed SAA3 IPEC-J2 cell line, was generated by transfecting a plasmid expressing pCAGGS-SAA3 through a recombination approach. A recombinant IPEC-J2-KD-SAA3 cell line, a SAA3 knock down IPEC-J2 cell line, was also constructed by being transfected with a vector pcDNA3.1-U6-shRNA that encompasses interference RNA. Both of the recombinant cell lines with over-expressing and silencing SAA3 were evaluated by western blot with antibodies against porcine SAA3. The confluent cultures of IPEC-J2, IPEC-J2-OE-SAA3, and IPEC-J2-KD-SAA3 were then inoculated with SADS-CoV-CH-FJWT-2018, a strain isolated in our lab from Fujian province in China, at an multiplicity of infection (MOI) of 1. Negative and positive controls were set up as well. The replication of SADS-CoV-CH-FJWT-2018 on the recombinant and normal IPEC-J2 cells was measured by TCID_50_ assays. SPSS software was used for statistical analysis (IBM, USA). Significant differences between groups were evaluated by using the Student's *t*-test. A threshold of *p* < 0.05 was regarded as a significant difference between two groups.

## Results

### SADS-CoV Infection in IPEC-J2 Cells and Vero-81 Cells

**Figure 1:**
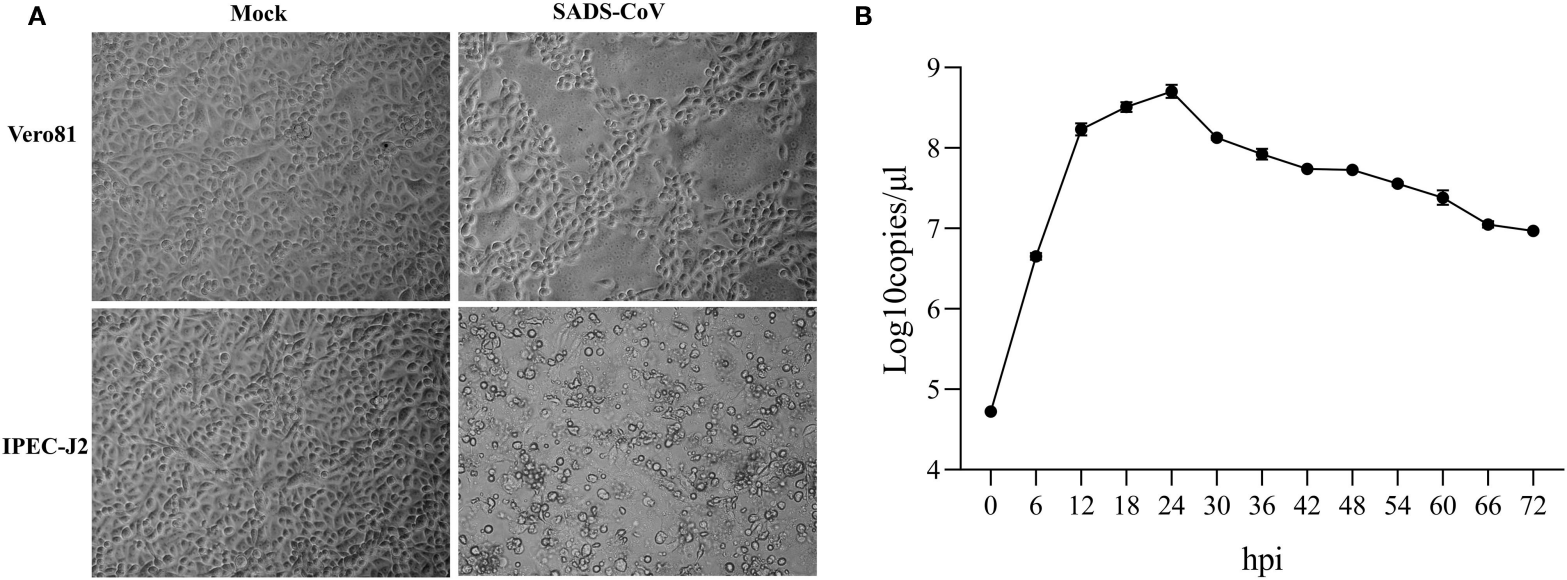
Cytopathic effects of SADS-CoV isolates in inoculated Vero 81 and IPEC-J2 cells **(A)** and One-step growth curve of SADS-CoV in IPEC-J2 cells **(B)** One-step growth curve of SADS-CoV in IPEC-J2 cells. Cells were inoculated with virus at an MOI of 1. Total virus titer was determined by qPCR-based infectivity assay at the indicated time intervals. Data shown are from experiments performed in triplicates, with error bars indicating standard deviations.

Vero 81 and IPEC-J2 cell lines were routinely employed to propagate the cell-adapted SADS-COV-CH-FJWT-2018. In Vero-81 cells, the CPE induced by SADS-CoV was characterized by cell fusion, while in IPEC-J2 cells, the CPE displayed as enlarged, rounded, and condensed granular particles (Figure 1A). To confirm the characteristics of SADS-CoV-CH-FJWT-2018 growth on IPEC-J2 cells, a growth curve was determined by quantifying the genomic RNA copies at different time intervals (0, 6, 12, 18, and up to 72 hpi) (Figure 1B). From 0 to 24 h, the viral titers were increased and reached a highest titer of 10^8.706^ copies/μl, and then decreased to 10^7.006^ copies/μl from 36 to 72 h. The results demonstrated that IPEC-J2 cells are susceptible and permissive to SADS-CoV-CH-FJWT-2018, and the cell line is a good candidate for SADS-CoV research.

### Expression Profiles of IPEC-J2 Cells Infected With SADS-COV-CH-FJWT-2018

The raw reads of RNA-Seq were quality controlled to ensure that all data were met the criteria for the whole transcriptomic analysis. A total of 132.80 Gb qualified bases were obtained with an average of 7.38 ± 0.70 Gb in each sample (Additional file 2: Table S2). The clean reads were then mapped to the reference pig genome (WASHUC2.69) in Ensemble using Hisat2 (v2.0.5), with an average of 94.84% qualified reads mapped reference sequences. A total of 24,676 genes were identified in which 23,677 were known genes and 999 novel genes (Additional file 3: Table S3). Of these known genes, 17,745 were protein coding genes, 4,096 were long non-coding RNA, 186 were miRNA, and 1,237 were pseudogenes or transcript pseudogenes.

### Differentially Expressed Genes Analyses

**Figure 2:**
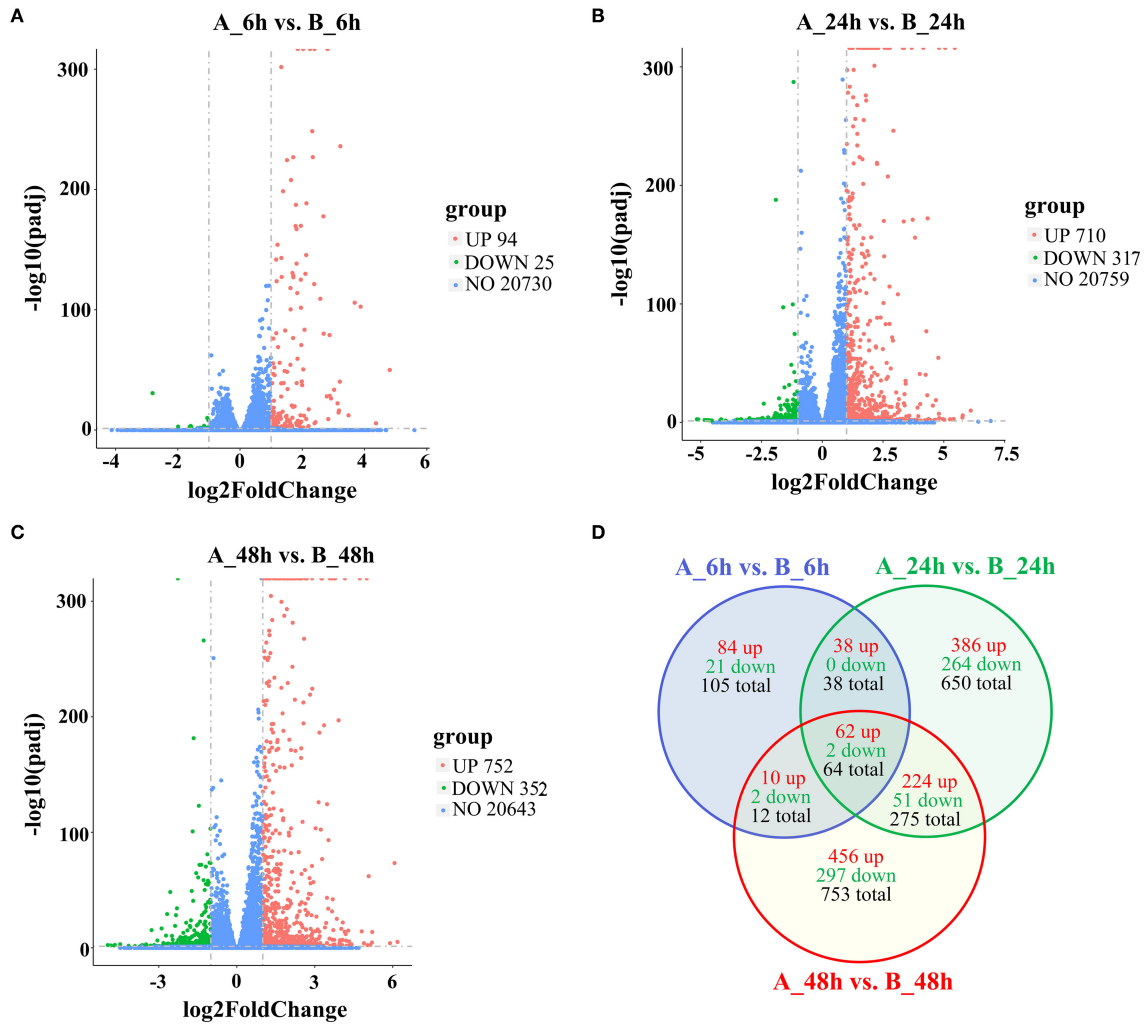
The differently expressed gene of volcano and Venn diagram analysis of up- and downregulated genes and shared genes. The vertical line of volcano diagram is the 2-fold expression difference threshold, and the horizontal line is the *p*_*adj*_-value = 0.05 threshold; the red, green, and blue points represent the upregulated, downregulated, and non-significant differentially expressed genes. **(A)** Infected vs. negative control at 6 hpi, **(B)** Infected vs. negative control at 24 hpi, **(C)** Infected vs. negative control at 48 hpi. **(D)** Venn diagram of DEGs distributions at 6, 24, and 48 hpi.

To identify the DEGs in response to SADS-CoV infection in IPEC-J2 cells, all the gene numbers were homogenized by an algorithm of Reads Per kb per Million reads (RPKM), and then DEGs were generated by horizontally compared between infected and negative control groups at 6, 24, and 48 hpi by DESeq2. A total of 1,897 DEGs were generated between the infected and uninfected samples at 6, 24, and 48 hpi, with 1,260 being upregulated and 637 downregulated (Table 1). The upregulated genes in 6, 24, and 48 dpi were 194, 710, and 752, respectively, while the downregulated genes were 25, 317, and 352, respectively (Figure 2A, Additional file 4: Table S4; Figure 2B, Additional file 5: Table S5; and Figure 2C, Additional file 6: Table S6). As the infection time passed, the DEGs, including the up- and downregulated DEGs, increased when compared to that of negative controls. At 6 hpi, a chemokine CXCL10 gene was the most upregulated gene, which was changed 28.05-fold; at 24 hpi, an interferon lambda-3-like gene LOC110255217 (NC_010448.4) was the most upregulated gene (69.07-fold); and, at 48 hpi, an interleukin receptor gene of IL17REL (NC_010447.5) was the most upregulated gene (73.01-fold). The most downregulated genes in groups of 6, 24, and 48 hpi were GTP-binding protein 6, LOC110257936 (NC_010462.3, 0.14-fold), novel gene 638 (NC_010449.5, 0.028-fold), and lncRNA LOC110255443 (NC_010451.4, 0.032-fold), respectively (Additional file 4: Table S4). There were also status-related changes in gene expression as shown in the Venn diagram (Figure 2D). Out of the 1,144 DEGs for both 6 hpi and 24 hpi samples between infected and uninfected cells, only 102 (8.92%) were common. There were 1,780 DEGs in both 24 hpi and 48 hpi samples, of which 339 (19.04%) were common. Only 64 DEGs were common when compared at 6, 24, and 48 hpi (Figure 2D).

**Table 1:** Summary of differentially expressed genes (DEGs) at log_2_|FC| ≥ 1 and *p*_*adj*_ < 0.05.

**Compared**	**Higher expression**	**No difference**	**Lower expression**	**DEG**
**groups**	**in infected**		**in infected**	
A_6h vs. B_6h	194	20,730	25	219
A_24h vs. B_24h	710	20,759	317	1,027
A_48h vs. B_48h	752	20,643	352	1,104

### GO Term Analysis

**Figure 3:**
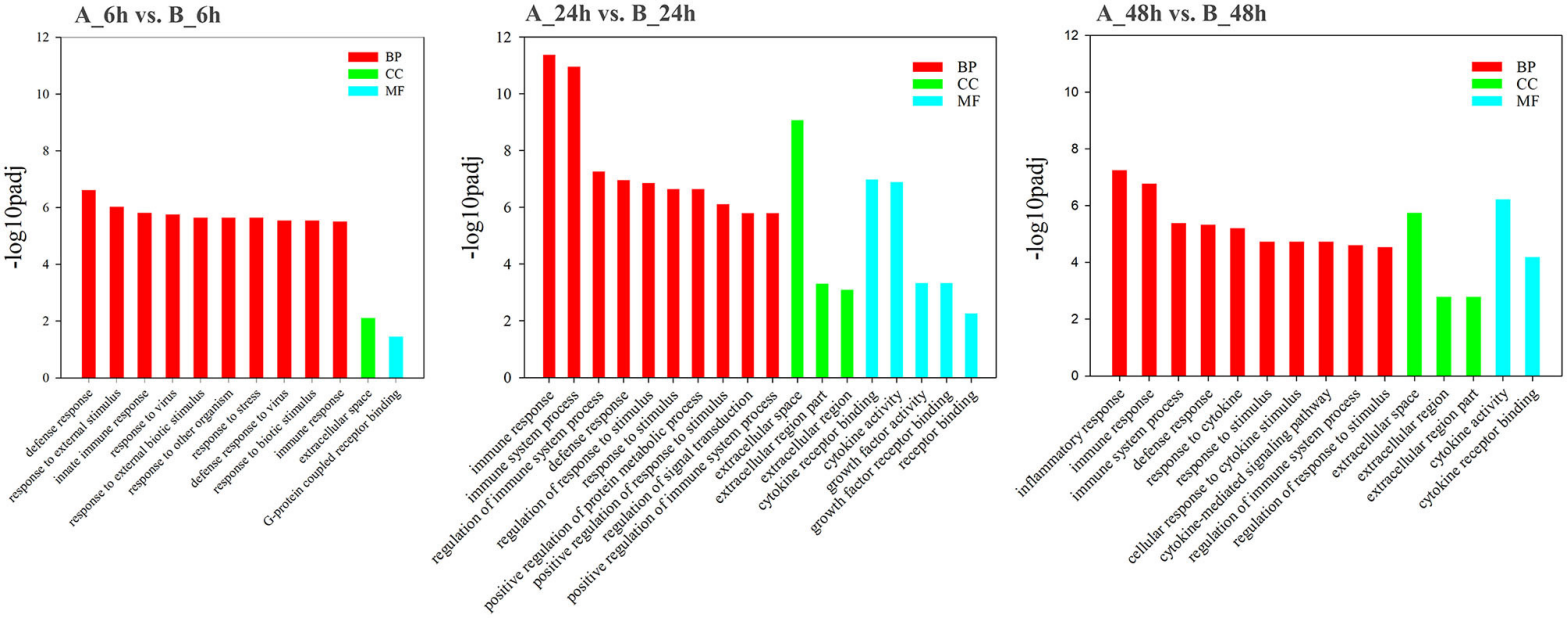
Functional map of differentially expressed genes enriched for GO terms. All categories were statistically significant (*p*_*adj*_ < 0.05). The bars represent the –log_10_*p*_*adj*_-value of the comparison of respective GO terms from infected and uninfected samples. Red, green, and blue bars indicated GO terms clustered in the biological process, cellular component, and molecular function terms, respectively.

**Figure 4:**
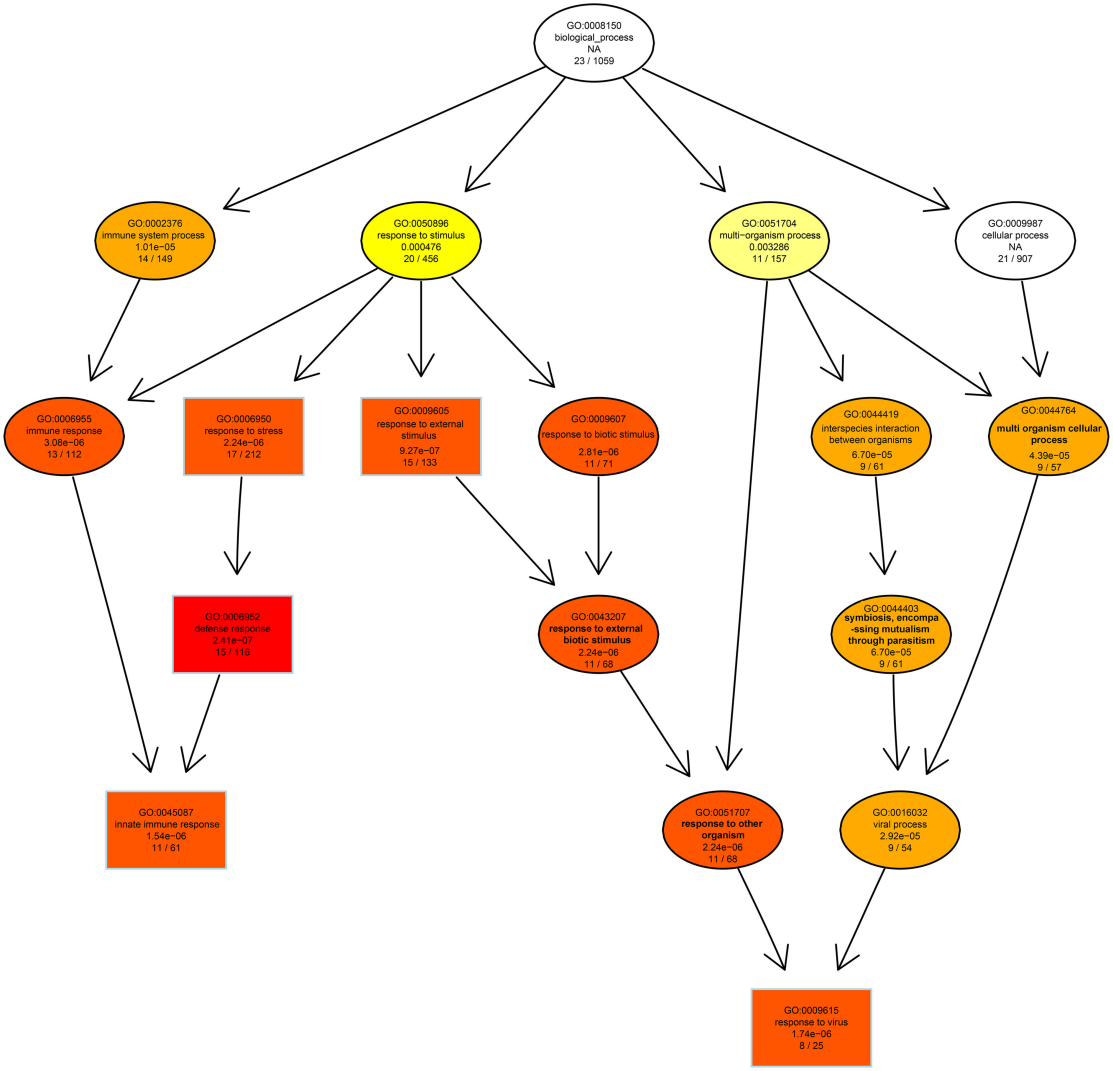
The directed acycline graph of the GO category in the biological process, indicating it is significantly enriched in infected samples at 6 hpi. Boxes and ellipses with colored background represent significantly enriched terms.

GO analysis was applied to determine the functions of the DEGs under the thresholds of *p* < 0.05 and FDR < 0.05. The 1,260 upregulated and 637 downregulated genes in the 6, 24, and 48 hpi stages were enriched in 79, 383, and 233 GO terms, respectively. Most of the DEGs were significantly enriched in biology process (BP), while only a few DEGs were enriched in cellular component (CC), and molecular function (MF) (Figure 3). Based on the GO terms, genes related to “immune system process,” “response to stimulus” and several other biological processes accounted for comparable percentage in 6 hpi samples (Additional file 7: Table S7). There were 76 DEGs in five GO terms related to “innate immune response” (GO: 0045087), such as immune “system process” (GO: 0002376), “response to stimulus” (GO: 0050896), “immune response” (GO: 0006955), “response to stress” (GO: 0006950), and “defense response” (GO: 0006952) were significantly enriched (*p*_*adj*_ < 0.05, Figure 4). A total of 115 DEGs in nine GO terms related to “response to virus” (GO: 0009615) were also significantly enriched in 6 hpi samples infected by SADS-CoV (Figure 4). This indicates that the infection of SADS-CoV could induce the innate immunity and antivirus response in the IPEC-J2 cells.

In 24 hpi samples, there were 5,144 genes annotated in 383 GO terms, 4,882 were upregulated, and 262 were downregulated; of the 4,882 upregulated genes, 4,703 (96.33%) were mapped into the BP terms, 111 (2.15%), and 75 (1.52%) were mapped into the CC and MF terms, respectively, (Additional file 8: Table S8). A total of 277 DEGs in seven GO terms were significantly enriched in “defense response” (GO: 0006952), “regulation of response to stimulus” (GO: 0048583), and “regulation of immune system process” (GO: 0002682) in 24 h post SADS-CoV infection (Additional file 9: Figure S1A). Furthermore, 113 DEGs in six GO terms were significantly enriched in protein binding, receptor binding, cytokine activity, cytokine receptor binding, growth factor activity, and growth factor receptor binding, which indicates the cytokine-cytokine receptor interactions were significantly upregulated in virus infected IPEC-J2 cells at 24 hpi (Additional file 9: Figure S1B).

In 48 hpi samples, there were 3,533 genes annotated in 232 GO terms, 3,213 were upregulated and 320 were downregulated; of the 3,213 upregulated genes, 3,081 (95.89%) were mapped into the BP terms, 101 (3.14%) and 31 (0.96%) were mapped into the CC and MF terms, respectively, (Additional file 10: Table S10). The most mapped GO item in all of the three time intervals (6, 24, and 48 hpi) was response to stimulus (GO: 0050896). In biological process category, 224 DEGs were significantly enriched in immune system processes GO terms and inflammatory response GO terms (Additional file 11: Figure S2A). In category of molecular function, 96 DEGs were mapped to protein- and receptor-binding, and cytokine-cytokine receptor binding GO terms (Additional file 11: Figure S2B). This indicates more intensify immune response, inflammation, and cytokines were induced as the infection was more serious in 48 h post SADS-CoV infection.

### KEGG Pathway Analysis

**Figure 5:**
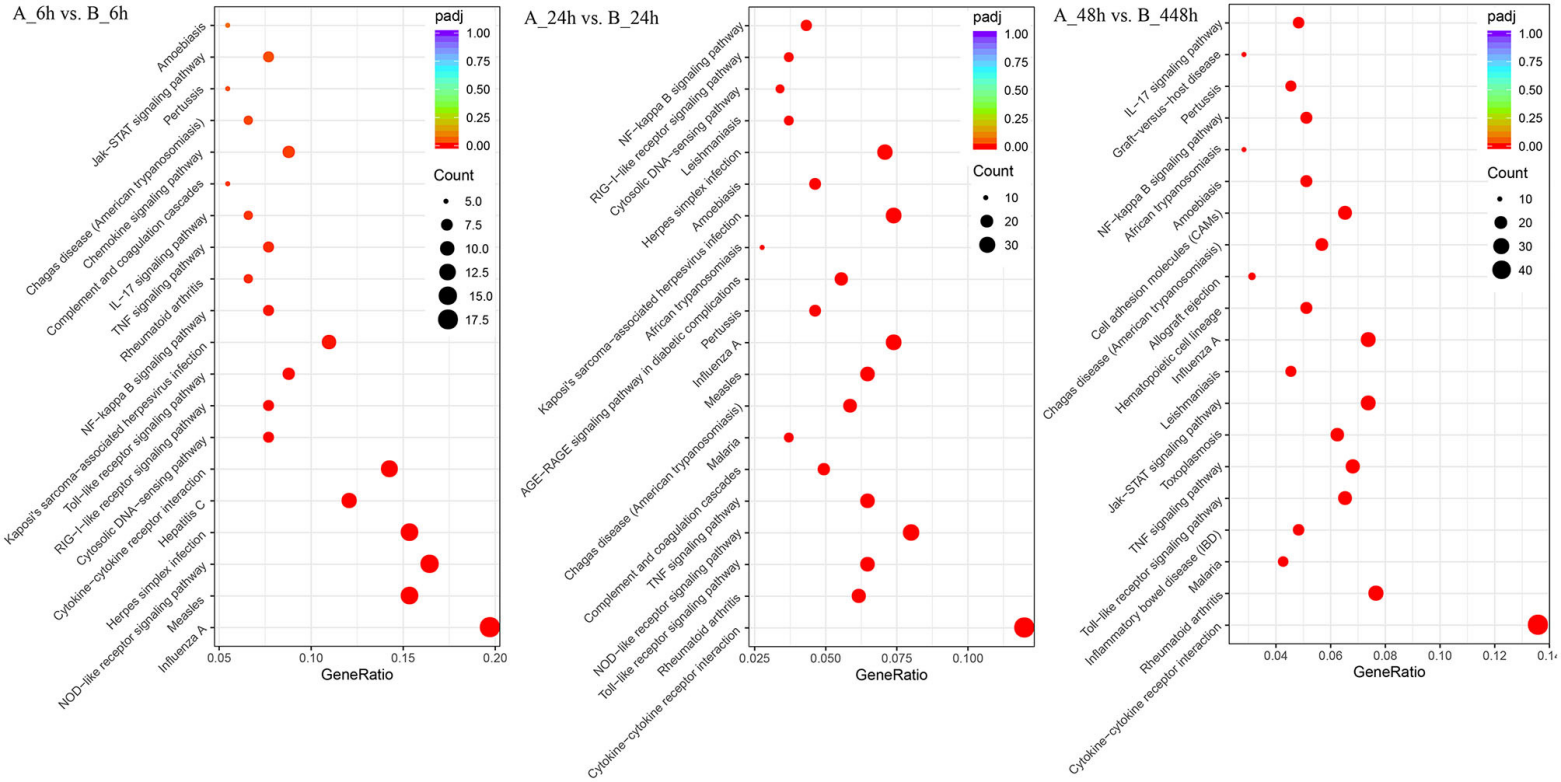
Different KEGG pathway gene enrichment statistics. The dot diameter indicates the number of differential genes; color depth indicates significance; abscissa indicates enrichment abundance; and the ordinate indicates different pathways.

The 1,897 DEGs were mapped to KEGG Ontology (KO), and 19, 38, and 52 KO categories were clustered in 6, 24, and 48 hpi samples. Noticeably, NOD-like receptor signaling pathway, Cytokine-cytokine receptor interaction, Toll-like receptor signaling pathway, TNF signaling pathway, and Chemokine signaling pathway were significantly enriched signaling pathways, which indicated the signals were promptly induced and intensified responded during the SADS-CoV infection (Figure 5). In 6 hpi samples, Infectious disease: viral (KEGG ID: ssc05164, ssc05162, ssc05168, ssc05160, and ssc05167), immune system, and signal transduction were the predominant terms (Additional file 12: Table S10). In the 24 hpi samples, 631 DEGs were mapped to categories of Environmental Information Processing, Human Diseases, and Organismal Systems in KO. Same as that of 6 hpi samples, Infectious disease: viral, immune system, and signal transduction were the predominant terms (Additional file 13: Table S11). In 48 hpi samples, 937 DEGs were clustered into 4 KO categories, Cellular Processes, Environmental Information Processing, Human Diseases, and Organismal Systems (Additional file 14: Table S12). Interestingly, the PI3K-Akt and Jak-STAT signaling pathways were significantly upregulated in SADS-CoV infected cells.

### Validation of DEGs in RNA-Seq by qRT-PCR

**Figure 6:**
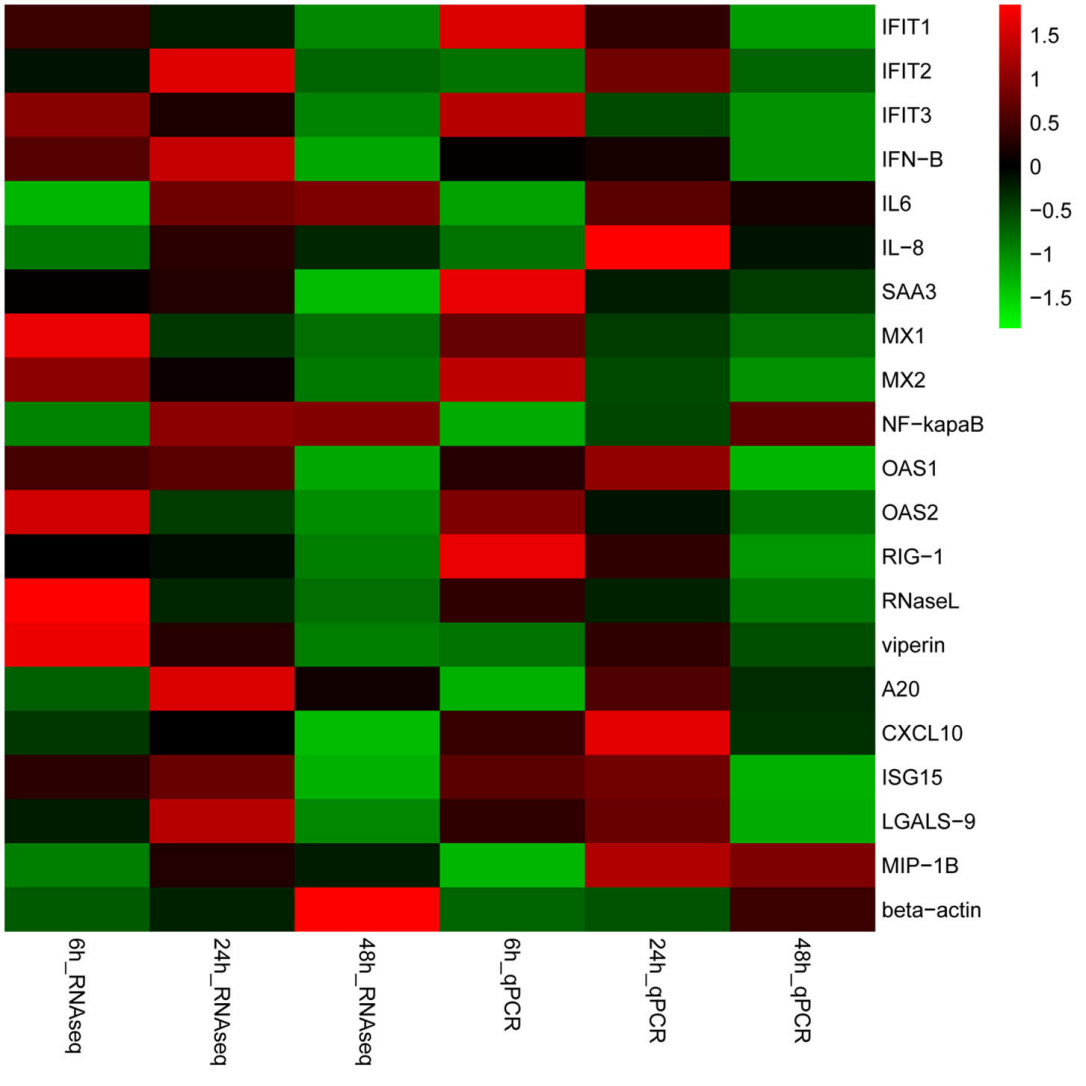
Validation of gene expression. Heat-maps of log_2_ transformed expression fold changes in SADS-CoV infected cells to time matched negative controls across the time points in hours using both RNA-Seq and quantitative real-time PCR (qPCR) methods. On the scale bar, red indicates upregulation, and green stands for downregulation of mRNA compared to mock-infected controls. Data are the medians of three independent biological replicates.

A total of 20 DEGs in the RNA-seq results were further verified by qRT-PCR. Our results shown that the mRNA expression level of the 20 randomly selected genes were in agreement with the expression of RNA-seq (Figure 6, Additional file 15: Table S13), which indicated that the RNA-seq data was reliable. Furthermore, the relative mRNA expression of these genes was represented in the Table S7. Overall, the validation of housekeeping and other selected genes by qRT-PCR demonstrated that the RNA-*Seq* results were reliable.

### The Effects of SAA-3 Protein on SADS-CoV Replication

**Figure 7:**
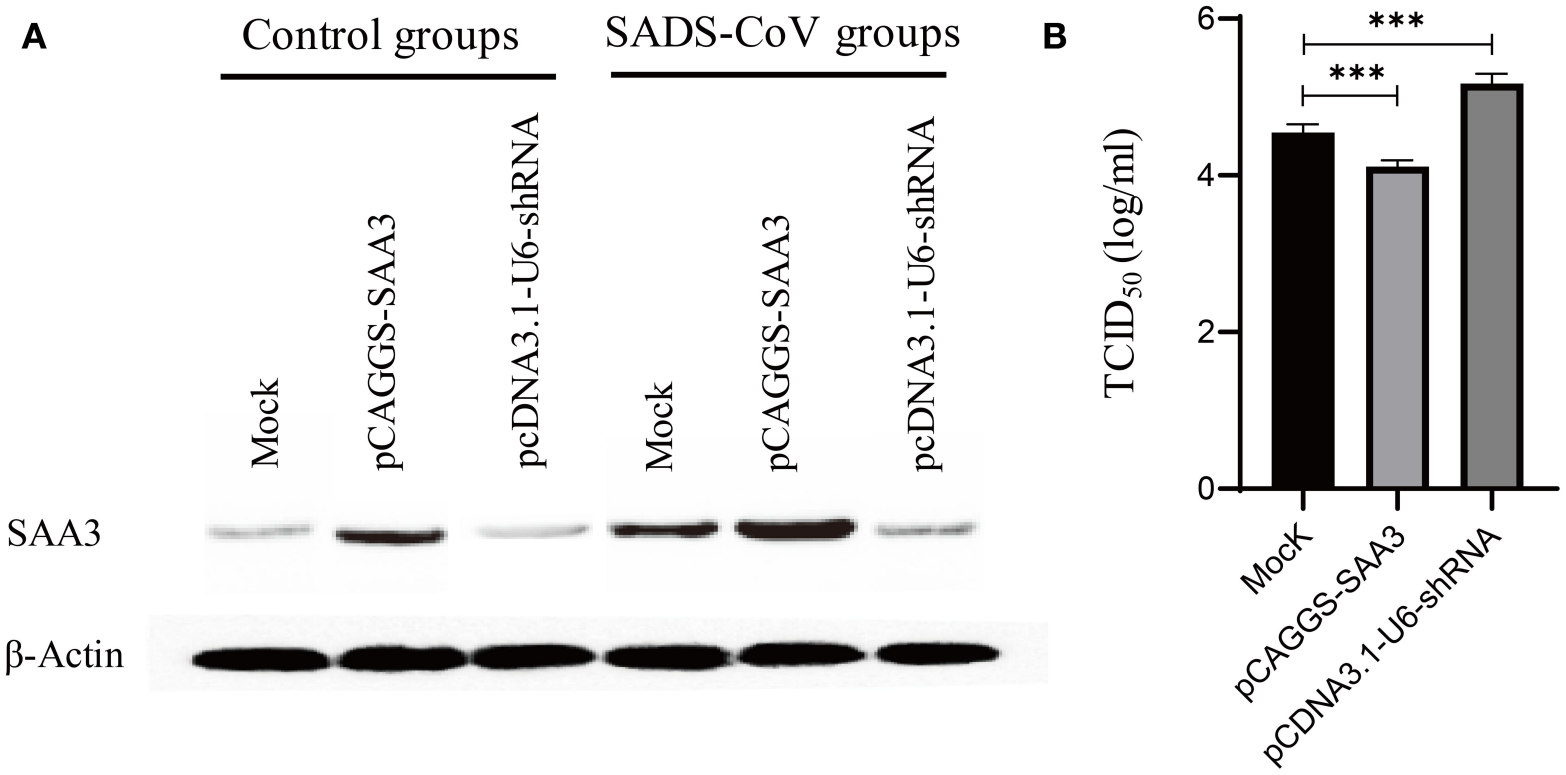
Measurement of SADS-CoV replication in recommended cells featured either overexpressed or silencing SAA3 protein gene. IPEC-J2 cells were transfected with an overexpressing/silencing plasmid pCAGGS-SAA3/pcDNA3.1-U6-shRNA or a mock vector and subsequently infected with SADS-CoV at an MOI of 1. The transfectants were harvested at 24 hpi and subjected to western blotting analysis **(A)** and a TCID_50_ assay **(B)**. **(A)** Expression/inhibition of SAA3 gene was detected by western blotting with an anti-SAA3 antibody. **(B)** SADS-CoV replication was titrated by a TCID_50_ assay. ***indicates the *p* value ≤ 0.001.

From the RNA-Seq dataset, SAA-3, an acute phase response protein, was significantly upregulated during the 6, 24, and 48 hpi samples (Additional files 4: Table S4, Additional files 5: Table S5, and Additional files 6: Table S6). To evaluate the effects of SAA3 on SADS-CoV infection, the recombinant IPEC-J2-OE-SAA3 cells with over-expression SAA3 and recombinant IPEC-J2-KD-SAA3 with interference of SAA3 were constructed. Western blot analysis showed that SAA3 was over expressed in IPEC-J2-OE-SAA3 cells, and obviously lower expressed in IPEC-J2-KD-SAA3 cells (Figure 7A). Then, the induction of SAA3 protein and TCID50 of SADS-CoV in IPEC-J2-OE-SAA3, IPEC-J2-KD-SAA3, and normal IPEC-J2 cells were compared. The results showed that SADS-CoV replication was significantly inhibited in the over-expression of SAA3. In contrast, shRNA interference silencing SAA3 gene promoted the expression of SADS-CoV in IPEC-J2 cells (Figure 7B).

## Discussion

Viral diarrhea caused by CoVs is still a severe problem in swine. Besides the known etiologies of PEDV and PDCoV, the emergence of SADS-CoV has made the situation more complicated (3, 8, 9, 33). Since SADS-CoV firstly was identified in early 2017 in Guangdong province in China, studies have mainly focused on the virus characterization and genetic evolution. The studies on the interactions between the hosts and SADS-CoV are lack. Thus, it is necessary to reveal the host-pathogen regulation, the SADS-CoV target genes, and the host immune response to the infection. To fill the gap of virus–host interactions, particularly the correlation between virus invasion and differential expressed host genes, the whole transcriptome IPEC-J2 cells during SADS-CoV infection were explored in this study.

We systematically analyzed the host defense and biological imperative modulations during infection of SADS-CoV in the annotated genes. We observed that many of the pathways and biological processes in SADS-CoV infected IPEC-J2 cells were significantly upregulated or downregulated. In particular, those genes, which were mainly involved in interactions of signal molecules, signal transduction, cell growth and death system, and immune system-related signaling pathways, were upregulated.

The innate immune response relies on recognition of evolutionarily conserved structures on pathogens, termed pathogen-associated molecular patterns (PAMPs), through a limited number of germ line-encoded pattern recognition receptors (PRRs), including Toll-like receptors (TLR), NOD-like receptors (NLR), and RIG-I-like receptors (RLRs) (34). The PAMP recognition of the extracellular RNA by PRRs (such as TLR2, TLR3, and TLR9 receptors) stimulated and signaled to the host the presence of infection and trigger inflammatory cytokines and antivirus responses by activating a multitude of intracellular signaling pathways, including adaptor molecules, kinases, and transcription factors. Then, the activation of gene expression and synthesis of a broad range of molecules, up-regulation of IL-6, CXCL2, IL-1β, IL-8, and other cytokines expression. For example, PEDV-infected porcine intestinal epithelial cells induces the rapid activation of the NF-κB pathway via the TLR family genes, while TLR2-, TLR3-, and TLR9-silenced genes can significantly inhibit the expression of NF-κB to promote the replication of PEDV (35). NF-κB is a transcription factor which regulates the expression of many factors that involve in immune system stimulation including a variety of pro-inflammatory cytokines, chemokines, and adhesion molecules (36). In this study, we demonstrated that the SADS-CoV infection significantly upregulated the expression of NFKBIA, NFKBIE, NFKBIB, NFKBID, and NFKBIZ, genes coding for inhibitors of κB (IκB) in IPEC-J2 cells during the three time points (6, 24, and 48 dpi), indicating the SADS-CoV infection could inhibit the expression of NF-κB and, thus, create a favorable milieu for SADS-CoV replication. NF-κB is a transcription factor responsible for modulating the expression of many genes involved in innate immunity, cell proliferation, differentiation, apoptosis, and metastasis. NF-κB interacts with IκB inhibitory proteins to regulate gene expression. In resting cells, NF-κB proteins are predominantly cytoplasmic, associating with members of the inhibitory IκB family. IκB proteins were originally thought to sequester NF-κB in the cytoplasm by masking its nuclear localization sequences (NLSs) (37). So, the upregulated of IκB in SADS-CoV infected cells might lead to the downregulated the activity of NF-κB, that inhibits the IFNs production. The antagonist of innate immune to promote viral survival/replication has been observed in many CoVs infections, including SARS-CoV, IBV, TGEV, PEDV, and PDCoV (38, 39). Thus, the results of our study are consistent with the previous studies. A recent study revealed that SADS-CoV interrupted poly (I:C)-induced phosphorylation and nuclear translocation of IRF3 and NF-κB (26). It might be another signal pathway that SADS-CoV antagonizes in terms of IFN production by upregulated IκB to inhibit the NF-κB pathway; further study is needed to illuminate the molecular mechanism.

SAA is one of the main acute phase proteins that are upregulated rapidly in response to infection, inflammation, and tissue damage in vertebrates (40, 41). Moreover, SAA induces a series of inflammatory mediators, including IL-1β, TNF-α, and IL-6 by binding to TLR2 and FPR2 receptors, and resulted in strong biological effects. In many virus infections, SAA is upregulated and plays distinct roles in the virus entry and replication in host cells (42). SAA interacts with hepatitis C virus particles to block virus entry into target cells (43). In present study, SADS-CoV replication was significantly inhibited in the over-expression of SAA3, while enhanced by silencing SAA3 in IPEC-J2 cell. Thus, SAA3 showed inhibition on SADS-CoV replication. The mechanism behind SAA3 suppression of the replication of SADS-CoV is still unknown, however, and further study is needed.

The replication of the virus depends on the host cell internal environments, so many viruses have evolved mechanisms that activate the host cell DNA damage signaling pathway, thereby blocking the cycle to produce the appropriate intracellular environment for proliferation (44–46). SARS coronavirus (SARS-CoV), murine hepatitis virus (MHV), avian infectious bronchitis virus (IBV), porcine transmissible gastroenteritis virus (TGEV), and PEDV can block the cell cycle (18, 21, 47, 48). According to the results in this study, proteins involved in functional cluster of cell cycle were significantly altered in SADS-CoV-infected cells when compared to the negative control.

In conclusion, this is the first report the interaction between SADS-CoV and IPEC-J2 cells by high-throughput RNA-Seq. Comprehensive functional analysis of host mRNA profiles revealed that SADS-CoV infection induced strong immune responses, including innate immunity, and cytokine-cytokine receptor interactions. We also identified the upregulated modulation activating the antiviral defenses of host cells through the elevated expression of immune-related genes and the changing the signaling pathways. The findings of this study could provide important insights into the modulation of host metabolism during SADS-CoV infection and have the potential to improve our understanding the pathogenesis of SADS-CoV.

## Data Availability Statement

All sample raw reads deposited at the Short Reads Archive (SRA) database belongs to the National Center for Biotechnology Information (NCBI) and are available under Bioproject ID PRJNA622652.

## Author Contributions

DS and YT designed the experiments. FZ, WY, and ZL performed the experiments. DS, YY, QW, and FZ carried out the data analysis. ZD and YC carried out the RNA-seq data evaluation. KL, TC, and FZ constructed the recombinant cell line. WY, ZL, YZ, and FZ carried out the virus propagation and titration. FZ, YY, ZD, DS, and YT prepared the manuscript. All authors read and approved the final manuscript.

## Conflict of Interest

The authors declare that the research was conducted in the absence of any commercial or financial relationships that could be construed as a potential conflict of interest.

## References

[1] Woo PC Lau SK Lam CS Lau CC Tsang AK Lau JH. Discovery of seven novel Mammalian and avian coronaviruses in the genus deltacoronavirus supports bat coronaviruses as the gene source of alphacoronavirus and betacoronavirus and avian coronaviruses as the gene source of gammacoronavirus and deltacoronavirus. J Virol. (2012) 86:3995–4008. 10.1128/JVI.06540-11 22278237 PMC3302495

[2] Wang Q Vlasova AN Kenney SP Saif LJ. Emerging and re-emerging coronaviruses in pigs. Curr Opin Virol. (2019) 34:39–49. 10.1016/j.coviro.2018.12.001 30654269 PMC7102852

[3] Zhou P Fan H Lan T Yang XL Shi WF Zhang W. Fatal swine acute diarrhoea syndrome caused by an HKU2-related coronavirus of bat origin. Nature. (2018) 556:255–8. 10.1038/s41586-018-0010-9 29618817 PMC7094983

[4] Zhou L Sun Y Lan T Wu RT Chen JW Wu ZX. Retrospective detection and phylogenetic analysis of swine acute diarrhea syndrome coronavirus in pigs in southern China. Transbound Emerg Dis. (2018) 66:687–95. 10.1111/tbed.13008 30171801 PMC7168530

[5] Gong L Li J Zhou Q Xu Z Chen L Zhang Y. A new bat-HKU2-like coronavirus in Swine, China, 2017. Emerg Infect Dis. (2017) 23:1607–9. 10.3201/eid2309.170915 28654418 PMC5572857

[6] Pan Y Tian X Qin P Wang B Zhao P Yang YL. Discovery of a novel swine enteric alphacoronavirus (SeACoV) in southern China. Vet Microbiol. (2017) 211:15–21. 10.1016/j.vetmic.2017.09.020 29102111 PMC7117260

[7] Li K Li H Bi Z Gu J Gong W Luo S. Complete genome sequence of a novel swine acute diarrhea syndrome coronavirus, CH/FJWT/2018, isolated in Fujian, China, in 2018. Microbiol Resour Announc. (2018) 7:e01259–18. 10.1128/MRA.01259-18 30533848 PMC6284080

[8] Zhou L Li QN Su JN Chen GH Wu ZX Luo Y. The re-emerging of SADS-CoV infection in pig herds in Southern China. Transbound Emerg Dis. (2019) 66:2180–3. 10.1111/tbed.13270 31207129 PMC7168562

[9] Xu Z Zhang Y Gong L Huang L Lin Y Qin J. Isolation and characterization of a highly pathogenic strain of Porcine enteric alphacoronavirus causing watery diarrhoea and high mortality in newborn piglets. Transbound Emerg Dis. (2019) 66:119–30. 10.1111/tbed.12992 30103259 PMC7168553

[10] Moon C Stappenbeck TS. Viral interactions with the host and microbiota in the intestine. Curr Opin Immunol. (2012) 24:405–10. 10.1016/j.coi.2012.05.002 22626624 PMC3423491

[11] Westermann AJ Vogel J. Host-pathogen transcriptomics by dual RNA-seq. Methods Mol Biol. (2018) 1737:59–75. 10.1007/978-1-4939-7634-8_4 29484587

[12] Depledge DP Srinivas KP Sadaoka T Bready D Mori Y Placantonakis DG. Direct RNA sequencing on nanopore arrays redefines the transcriptional complexity of a viral pathogen. Nat Commun. (2019) 10:754. 10.1038/s41467-019-08734-9 30765700 PMC6376126

[13] Foulger RE Osumi-Sutherland D McIntosh BK Hulo C Masson P Poux S. Representing virus-host interactions and other multi-organism processes in the gene ontology. BMC Microbiol. (2015) 15:146. 10.1186/s12866-015-0481-x 26215368 PMC4517558

[14] Salguero FJ Frossard JP Rebel JM Stadejek T Morgan SB Graham SP. Host-pathogen interactions during porcine reproductive and respiratory syndrome virus 1 infection of piglets. Virus Res. (2015) 202:135–43. 10.1016/j.virusres.2014.12.026 25559070 PMC7172408

[15] Sun P Fahd Q Li Y Sun Y Li J Qaria MA. Transcriptomic analysis of small intestinal mucosa from porcine epidemic diarrhea virus infected piglets. Microb Pathog. (2019) 132:73–9. 10.1016/j.micpath.2019.04.033 31026494 PMC7125762

[16] Wang X Jia Y Ren J Huo N Liu H Xiao S. Newcastle disease virus non-structural V protein upregulates SOCS3 expression to facilitate viral replication depending on the MEK/ERK pathway. Front Cell Infect Microbiol. (2019) 9:317. 10.3389/fcimb.2019.00317 31552199 PMC6748215

[17] Zhao S Gao J Zhu L Yang Q. Transmissible gastroenteritis virus and porcine epidemic diarrhoea virus infection induces dramatic changes in the tight junctions and microfilaments of polarized IPEC-J2 cells. Virus Res. (2014) 192:34–45. 10.1016/j.virusres.2014.08.014 25173696 PMC7114495

[18] Xu X Zhang H Zhang Q Huang Y Dong J Liang Y. Porcine epidemic diarrhea virus N protein prolongs S-phase cell cycle, induces endoplasmic reticulum stress, and upregulates interleukin-8 expression. Vet Microbiol. (2013) 164:212–21. 10.1016/j.vetmic.2013.01.034 23562137 PMC7117426

[19] Yuan X Wu J Shan Y Yao Z Dong B Chen B. SARS coronavirus 7a protein blocks cell cycle progression at G0/G1 phase via the cyclin D3/pRb pathway. Virology. (2006) 346:74–85. 10.1016/j.virol.2005.10.015 16303160 PMC7111786

[20] Chen CJ Sugiyama K Kubo H Huang C Makino S. Murine coronavirus non-structural protein p28 arrests cell cycle in G0/G1 phase. J Virol. (2004) 78:10410–9. 10.1128/JVI.78.19.10410-10419.2004 15367607 PMC516409

[21] Li FQ Tam JP Liu DX. Cell cycle arrest and apoptosis induced by the coronavirus infectious bronchitis virus in the absence of p53. Virology. (2007) 365:435–45. 10.1016/j.virol.2007.04.015 17493653 PMC7103336

[22] Yuan X Yao Z Wu J Zhou Y Shan Y Dong B. G1 phase cell cycle arrest induced by SARS-CoV 3a protein via the cyclin D3/pRb pathway. Am J Respir Cell Mol Biol. (2007) 37:9–19. 10.1165/rcmb.2005-0345RC 17413032

[23] Diez-Fuertes F de La Torre-Tarazona HE Calonge E Pernas M Alonso-Socas M Capa L. Transcriptome sequencing of peripheral blood mononuclear cells from elite controller-long term non progressors. Sci Rep. (2019) 9:14265. 10.1038/s41598-019-50642-x 31582776 PMC6776652

[24] Mohammadi P Castel SE Cummings BB Einson J Sousa C Hoffman P. Genetic regulatory variation in populations informs transcriptome analysis in rare disease. Science. (2019) 366:351–6. 10.1126/science.aay0256 31601707 PMC6814274

[25] Sun D Zhang X Zhang Q Ji X Jia Y Wang H. Comparative transcriptome profiling uncovers a Lilium regale NAC transcription factor, LrNAC35, contributing to defence response against cucumber mosaic virus and tobacco mosaic virus. Mol Plant Pathol. (2019) 20:1662–81. 10.1111/mpp.12868 31560826 PMC6859495

[26] Zhou Z Sun Y Yan X Tang X Li Q Tan Y. Swine acute diarrhea syndrome coronavirus (SADS-CoV) antagonizes interferon-beta production via blocking IPS-1 and RIG-I. Virus Res. (2019) 278:197843. 10.1016/j.virusres.2019.197843 31884203 PMC7114844

[27] Jung K Miyazaki A Hu H Saif LJ. Susceptibility of porcine IPEC-J2 intestinal epithelial cells to infection with porcine deltacoronavirus (PDCoV) and serum cytokine responses of gnotobiotic pigs to acute infection with IPEC-J2 cell culture-passaged PDCoV. Vet Microbiol. (2018) 221:49–58. 10.1016/j.vetmic.2018.05.019 29981708 PMC7117386

[28] Li K Li H Bi Z Song D Zhang F Lei D. Significant inhibition of re-emerged and emerging swine enteric coronavirus *in vitro* using the multiple shRNA expression vector. Antiviral Res. (2019) 166:11–8. 10.1016/j.antiviral.2019.03.010 30905822 PMC7113732

[29] Lin H Chen L Gao L Yuan X Ma Z Fan H. Epidemic strain YC2014 of porcine epidemic diarrhea virus could provide piglets against homologous challenge. Virol J. (2016) 13:68. 10.1186/s12985-016-0529-z 27103490 PMC4840883

[30] Purandare SR Bickel RD Jaquiery J Rispe C Brisson JA. Accelerated evolution of morph-biased genes in pea aphids. Mol Biol Evol. (2014) 31:2073–83. 10.1093/molbev/msu149 24770714 PMC4104313

[31] Tarazona S Garcia-Alcalde F Dopazo J Ferrer A Conesa A. Differential expression in RNA-seq: a matter of depth. Genome Res. (2011) 21:2213–23. 10.1101/gr.124321.111 21903743 PMC3227109

[32] Kanehisa M Goto S. KEGG: kyoto encyclopedia of genes and genomes. Nucleic Acids Res. (2000) 28:27–30. 10.1093/nar/28.1.27 10592173 PMC102409

[33] Yang YL Liang QZ Xu SY Mazing E Xu GH Peng L. Characterization of a novel bat-HKU2-like swine enteric alphacoronavirus (SeACoV) infection in cultured cells and development of a SeACoV infectious clone. Virology. (2019) 536:110–8. 10.1016/j.virol.2019.08.006 31419711 PMC7112019

[34] Gao S Wang Z Jiang H Sun J Diao Y Tang Y. Transcriptional analysis of host responses related to immunity in chicken spleen tissues infected with reticuloendotheliosis virus strain SNV. Infect Genet Evol. (2019) 74:103932. 10.1016/j.meegid.2019.103932 31228642

[35] Cao L Ge X Gao Y Ren Y Ren X Li G. Porcine epidemic diarrhea virus infection induces NF-κB activation through the TLR2, TLR3 and TLR9 pathways in porcine intestinal epithelial cells. J Gen Virol. (2015) 96 (Pt. 7):1757–67. 10.1099/vir.0.000133 25814121

[36] Geng H Wittwer T Dittrich-Breiholz O Kracht M Schmitz ML. Phosphorylation of NF-kappaB p65 at Ser468 controls its COMMD1-dependent ubiquitination and target gene-specific proteasomal elimination. Embo Rep. (2009) 10:381–6. 10.1038/embor.2009.10 19270718 PMC2672889

[37] Moynagh PN. The NF-kB pathway. J Cell Sci. (2005) 118 (Pt. 20):4589–92. 10.1242/jcs.02579 16219681

[38] Channappanavar R Perlman S. Pathogenic human coronavirus infections: causes and consequences of cytokine storm and immunopathology. Semin Immunopathol. (2017) 39:529–39. 10.1007/s00281-017-0629-x 28466096 PMC7079893

[39] Zhang Q Yoo D. Immune evasion of porcine enteric coronaviruses and viral modulation of antiviral innate signaling. Virus Res. (2016) 226:128–41. 10.1016/j.virusres.2016.05.015 27212682 PMC7111337

[40] Zhang Y Zhang J Sheng H Li H Wang R. Acute phase reactant serum amyloid A in inflammation and other diseases. Adv Clin Chem. (2019) 90:25–80. 10.1016/bs.acc.2019.01.002 31122611

[41] Zhou J Sheng J Fan Y Zhu X Tao Q He Y. Association between serum amyloid A levels and cancers: a systematic review and meta-analysis. Postgrad Med J. (2018) 94:499–507. 10.1136/postgradmedj-2018-136004 30341230

[42] Lannergard A Larsson A Kragsbjerg P Friman G. Correlations between serum amyloid A protein and C-reactive protein in infectious diseases. Scand J Clin Lab Invest. (2003) 63:267–72. 10.1080/00365510308550 12940634

[43] Cai Z Cai L Jiang J Chang KS van der Westhuyzen DR Luo G. Human serum amyloid A protein inhibits hepatitis C virus entry into cells. J Virol. (2007) 81:6128–33. 10.1128/JVI.02627-06 17329325 PMC1900255

[44] Luftig MA. Viruses and the DNA damage response: activation and antagonism. Annu Rev Virol. (2014) 1:605–25. 10.1146/annurev-virology-031413-085548 26958736

[45] Hollingworth R Grand RJ Modulation of DNA damage and repair pathways by human tumour viruses. Viruses. (2015) 7:2542–91. 10.3390/v7052542 26008701 PMC4452920

[46] Yoshinaga N Shindo K Matsui Y Takiuchi Y Fukuda H Nagata K. A screening for DNA damage response molecules that affect HIV-1 infection. Biochem Biophys Res Commun. (2019) 513:93–8. 10.1016/j.bbrc.2019.03.168 30935695

[47] Sun P Wu H Huang J Xu Y Yang F Zhang Q. Porcine epidemic diarrhea virus through p53-dependent pathway causes cell cycle arrest in the G0/G1 phase. Virus Res. (2018) 253:1–11. 10.1016/j.virusres.2018.05.019 29800601 PMC7114671

[48] Vogt A Scull MA Friling T Horwitz JA Donovan BM Dorner M. Recapitulation of the hepatitis C virus life-cycle in engineered murine cell lines. Virology. (2013) 444:1–11. 10.1016/j.virol.2013.05.036 23777661 PMC3755106

