# Supplementary material for: RNA-Seq-Based Whole Transcriptome Analysis of IPEC-J2 Cells During Swine Acute Diarrhea Syndrome Coronavirus Infection
Source: Front Vet Sci. 2020 Aug 13;7:492. doi: 10.3389/fvets.2020.00492 (PMC7438718; doi:10.3389/fvets.2020.00492)
Supplement: Supplementary file 1 [file Table_1.docx]

Table S1 Primers used to verify the selected genes in the RNA-Seq results in this study

| Gene | Primer sequence( 5’-3’) | Tm (℃) | Product size (bp) | Genebank accession No. of Reference sequence |
| --- | --- | --- | --- | --- |
| IFIT1 | F: TACATTTCCACTATGGCCGAT | 56.86 | 251 | HQ679904 |
|  | R: GGCCTGCTCATAATACTCCA | 56.77 |  |  |
| IFIT2 | F: AGGAACTAATAGGACACGCTCT | 58.37 | 206 | XM_005671264 |
|  | R: CTGAAAGTTGCCGTATCGCAG | 59.94 |  |  |
| IFIT3 | F: GCAGCCAAATTTTACCGAGT | 56.99 | 211 | HQ200930 |
|  | R: AGTCCTTGGCATATTTCCGTA | 56.79 |  |  |
| IFN-β | F: AGTTGCCTGGGACTCCTCAA | 60.77 | 60 | GQ415073 |
|  | R: CCTCAGGGACCTCAAAGTTCAT | 59.69 |  |  |
| IL6 | F: CTGGCAGAAAACAACCTGAACC | 60.22 | 94 | NM_214399 |
|  | R: TGATTCTCATCAAGCAGGTCTCC | 60.12 |  |  |
| IL8 | F: AGTTTTCCTGCTTTCTGCAGCT | 61.01 | 72 | AB057440 |
|  | R: TGGCATCGAAGTTCTGCACT | 59.96 |  |  |
| SAA3 | F: AGCGATGCCAGAGAGAATGT | 59.17 | 144 | XM_013994502 |
|  | R: ACGAGGTCTGAAGTGGTTGG | 59.61 |  |  |
| MX1 | F: GGCGTGGGAATCAGTCATG | 58.6 | 81 | AH015318 |
|  | R: AGGAAGGTCTATGAGGGTCAGA | 59.41 |  |  |
| MX2 | F: GGAAATACGCAAAGCCCA | 55.64 | 209 | AB854078 |
|  | R: TCTGCTGCTCCTGGATGTA | 57.69 |  |  |
| NF-kB | F: CGAGGAAATACCCCTCTACACCT | 60.94 | 196 | FN421469 |
|  | R: CAGCACCCAAAGACACCAAC | 59.61 |  |  |
| OAS1 | F: ATGTTTCCGAACGCAGGT | 57.21 | 119 | NM_214303 |
|  | R: AGGAGCCACCCTTCACAACT | 61.06 |  |  |
| OAS2 | F: CAACTCTGATGGTTCCCTTG | 56.03 | 204 | XM_021072182 |
|  | R: ATGCTCTGCTCTTTAGCGA | 56.54 |  |  |
| RIG-I | F: CCCTGGTTTAGGGACGATGA | 58.8 | 62 | KC011279 |
|  | R: GTCGGGCCCTTGTTGTTTTT | 59.54 |  |  |
| RNase L | F: GCAGCCGAGCCAACGATA | 60.2 | 57 | NM_001097512 |
|  | R: AGCTCCCGTCGCTCTCACT | 62.93 |  |  |
| TGF-β3 | F: AGCACAATGATCTGGCCGTT | 60.32 | 354 | XM_013978322 |
|  | R: GCTGAAAGGTGTGACACGGA | 60.53 |  |  |
| viperin | F: AGAGCCGTTTATCCACGAC | 57.26 | 168 | NM_213817 |
|  | R: TCACAGGAGATGGCAAGAA | 55.93 |  |  |
| A20 | F: CCTCCCTGGAAAGCCAGAA | 58.92 | 60 | NM_001267890 |
|  | R: GTGCCACAAGCTTCCTCACTT | 61.09 |  |  |
| CXCL10 | F: ACTGTTCGCTGTACCTGCAT | 59.68 | 233 | EU364898 |
|  | R: GCTTCTCTCTGTGTTCGAGGA | 59.46 |  |  |
| ISG15 | F: GTGGGTCTCTGAAGCTTTGC | 59.12 | 240 | NM_001128469 |
|  | R: TGGGTAGGGAACTGAAGGTG | 58.64 |  |  |
| LGALS-9 | F: CCCATCCAGCCCACCTTCTCC | 64.56 | 83 | FJ428535 |
|  | R: GGTTTCGGTTTGCGCCCTTTG | 63.44 |  |  |
| MIP-1β | F: CTCTCCTCCAGCAAGACCA | 58.32 | 299 | NM_213779 |
|  | R: GCTCAGTTCAGTTCCAAGTCA | 58.16 |  |  |
| Beta-actin | F: GGTGGGTATGGGTCAGAAAG | 57.58 | 117 | AF054837 |
|  | R: TCCATGTCGTCCCAGTTGGT | 61.13 |  |  |
| GAPDH | F: AAGGTCGGAGTGAACGGATTTG | 60.87 | 173 | NM_001206359 |
|  | R: GCCTTGACTGTGCCGTGGAAC | 64.32 |  |  |
