# Supplementary material for: RNA-Seq-Based Whole Transcriptome Analysis of IPEC-J2 Cells During Swine Acute Diarrhea Syndrome Coronavirus Infection
Source: Front Vet Sci. 2020 Aug 13;7:492. doi: 10.3389/fvets.2020.00492 (PMC7438718; doi:10.3389/fvets.2020.00492)
Supplement: Supplementary file 2 [file Table_2.docx]

Table S2 Reads counts and quality of samples used in RNA-Seq

| Sample | Raw Reads No. | Clean Reads No. | Clean Bases (Gb) | Clean Reads Q20 (%) | Mapped reads  No. (percent %) | Unique mapped reads No. (percent %) | Multi mapped reads  No. (percent %) |
| --- | --- | --- | --- | --- | --- | --- | --- |
| A1_6h | 50,678,496 | 49,017,754 | 7.35 | 97.36 | 46,499,013(94.86) | 44,348,736(90.47) | 2,150,277(4.39) |
| A2_6h | 43,374,758 | 41,694,528 | 6.25 | 97.48 | 39,566,837(94.9) | 37,712,597(90.45) | 1,854,240(4.45) |
| A3_6h | 61,738,028 | 60,034,680 | 9.01 | 97.56 | 57,175,243(95.24) | 54,435,783(90.67) | 2,739,460(4.56) |
| B1_6h | 54,350,284 | 52,978,932 | 7.95 | 97.45 | 50,431,307(95.19) | 48,276,740(91.12) | 2,154,567(4.07) |
| B2_6h | 53,725,358 | 52,186,684 | 7.83 | 97.21 | 49,513,588(94.88) | 47,447,810(90.92) | 2,065,778(3.96) |
| B3_6h | 43,446,734 | 42,176,418 | 6.33 | 97.7 | 40,286,607(95.52) | 38,633,499(91.6) | 1,653,108(3.92) |
| A1_24h | 51,742,184 | 50,290,180 | 7.54 | 97.6 | 47,607,824(94.67) | 45,363,549(90.2) | 2,244,275(4.46) |
| A2_24h | 49,720,724 | 47,648,684 | 7.15 | 97.49 | 45,029,686(94.5) | 42,873,218(89.98) | 2,156,468(4.53) |
| A3_24h | 52,781,294 | 51,032,186 | 7.65 | 97.61 | 48,217,076(94.48) | 45,942,294(90.03) | 2,274,782(4.46) |
| B1_24h | 53,791,224 | 52,446,986 | 7.87 | 97.4 | 49,593,118(94.56) | 47,020,396(89.65) | 2,572,722(4.91) |
| B2_24h | 54,215,894 | 52,684,740 | 7.90 | 97.68 | 49,941,891(94.79) | 47,264,888(89.71) | 2,677,003(5.08) |
| B3_24h | 48,522,078 | 47,028,938 | 7.05 | 97.7 | 44,536,281(94.7) | 42,167,756(89.66) | 2,368,525(5.04) |
| A1_48h | 52,615,516 | 51,270,374 | 7.69 | 97.82 | 48,459,400(94.52) | 46,713,357(91.11) | 1,746,043(3.41) |
| A2_48h | 45,326,232 | 43,888,214 | 6.58 | 97.88 | 41,637,442(94.87) | 40,147,468(91.48) | 1,489,974(3.39) |
| A3_48h | 49,164,958 | 47,388,876 | 7.11 | 97.76 | 44,931,767(94.82) | 43,292,594(91.36) | 1,639,173(3.46) |
| B1_48h | 48,309,616 | 46,001,720 | 6.90 | 97.81 | 43,714,873(95.03) | 41,910,482(91.11) | 1,804,391(3.92) |
| B2_48h | 55,100,960 | 53,362,044 | 8.00 | 97.47 | 505,791,94(94.78) | 48,495,960(90.88) | 2,083,234(3.9) |
| B3_48h | 45,623,424 | 44,278,724 | 6.64 | 97.57 | 41,980,377(94.81) | 40,241,794(90.88) | 1,738,583(3.93) |
