# Supplementary material for: RNA-Seq-Based Whole Transcriptome Analysis of IPEC-J2 Cells During Swine Acute Diarrhea Syndrome Coronavirus Infection
Source: Front Vet Sci. 2020 Aug 13;7:492. doi: 10.3389/fvets.2020.00492 (PMC7438718; doi:10.3389/fvets.2020.00492)
Supplement: Supplementary file 3 [file Table_3.docx]

**Table S3 Statistics of expressed genes in RNA-*Seq* in this study**

| Sample | Genes Num | Ratio | Group | Genes Num | Ratio |
| --- | --- | --- | --- | --- | --- |
| A1_6h | 19208 | 77.84% | A_6h | 21082 | 85.44% |
| A2_6h | 18824 | 76.28% |  |  |  |
| A3_6h | 19493 | 79.00% |  |  |  |
| B1_6h | 19129 | 77.52% | B_6h | 20932 | 84.83% |
| B2_6h | 18980 | 76.92% |  |  |  |
| B3_6h | 18781 | 76.11% |  |  |  |
| A1_24h | 19655 | 79.65% | A_24h | 21630 | 87.66% |
| A2_24h | 19672 | 79.72% |  |  |  |
| A3_24h | 19807 | 80.27% |  |  |  |
| B1_24h | 20122 | 81.54% | B_24h | 21927 | 88.86% |
| B2_24h | 20131 | 81.58% |  |  |  |
| B3_24h | 19942 | 80.82% |  |  |  |
| A1_48h | 19908 | 80.68% | A_48h | 21712 | 87.99% |
| A2_48h | 19542 | 79.19% |  |  |  |
| A3_48h | 19518 | 79.10% |  |  |  |
| B1_48h | 19814 | 80.30% | B_48h | 21786 | 88.29% |
| B2_48h | 19962 | 80.90% |  |  |  |
| B3_48h | 19762 | 80.09% |  |  |  |
| Total | 24676 |  |  | 24676 |  |
