# Supplementary material for: RNA-Seq-Based Whole Transcriptome Analysis of IPEC-J2 Cells During Swine Acute Diarrhea Syndrome Coronavirus Infection
Source: Front Vet Sci. 2020 Aug 13;7:492. doi: 10.3389/fvets.2020.00492 (PMC7438718; doi:10.3389/fvets.2020.00492)
Supplement: Supplementary file 13 [file Table_13.docx]

Table S13 Verification of RNA-Seq results

| Selected gene | FoldChange of genes of SADS-CoV vs mock | | | | | |
| --- | --- | --- | --- | --- | --- | --- |
|  | 6h | | 24h | | 48h | |
|  | RNA-Seq | qPCR | RNA-Seq | qPCR | RNA-Seq | qPCR |
| IFIT1 | 2.338 | 3.086 | 1.811 | 2.278 | 0.755 | 0.186 |
| IFIT2 | 2.687 | 1.767 | 4.158 | 3.582 | 1.954 | 1.967 |
| IFIT3 | 2.391 | 2.569 | 1.895 | 1.274 | 0.665 | 0.465 |
| IFN-B | 3.839 | 3.497 | 4.256 | 3.579 | 2.638 | 2.744 |
| IL6 | 0.453 | 0.695 | 1.979 | 1.911 | 2.020 | 1.681 |
| IL-8 | 1.820 | 1.898 | 3.310 | 4.542 | 2.658 | 2.850 |
| SAA3 | 3.224 | 3.937 | 3.316 | 3.082 | 2.423 | 2.977 |
| MX1 | 2.251 | 1.770 | 0.973 | 0.946 | 0.383 | 0.373 |
| MX2 | 2.320 | 2.486 | 1.742 | 1.230 | 0.850 | 0.485 |
| NF-kappaB | 0.589 | -0.170 | 1.581 | 0.912 | 1.544 | 1.454 |
| OAS1 | 1.839 | 1.723 | 1.908 | 2.094 | 0.606 | 0.406 |
| OAS2 | 1.855 | 1.669 | 1.140 | 1.282 | 0.825 | 0.930 |
| RIG-1 | 1.796 | 2.585 | 1.737 | 1.986 | 1.128 | 0.963 |
| RNaseL | 0.750 | 0.530 | 0.405 | 0.412 | 0.267 | 0.242 |
| viperin | 2.815 | 1.825 | 2.308 | 2.336 | 1.778 | 1.947 |
| A20 | 1.167 | 0.627 | 2.225 | 1.839 | 1.636 | 1.387 |
| CXCL10 | 4.811 | 5.265 | 5.058 | 5.943 | 4.161 | 4.823 |
| ISG15 | 2.028 | 2.215 | 2.285 | 2.332 | 0.312 | 0.362 |
| LGALS-9 | 0.968 | 1.184 | 1.465 | 1.311 | 0.574 | 0.351 |
| MIP-1B | 1.829 | 1.116 | 3.042 | 3.812 | 2.623 | 3.545 |
| beta-actin | -0.115 | -0.001 | 0.334 | 0.153 | 0.777 | 0.515 |
