# Supplementary figures and images for: RNA-Seq-Based Whole Transcriptome Analysis of IPEC-J2 Cells During Swine Acute Diarrhea Syndrome Coronavirus Infection
Source: Front Vet Sci. 2020 Aug 13;7:492. doi: 10.3389/fvets.2020.00492 (PMC7438718; doi:10.3389/fvets.2020.00492)

A

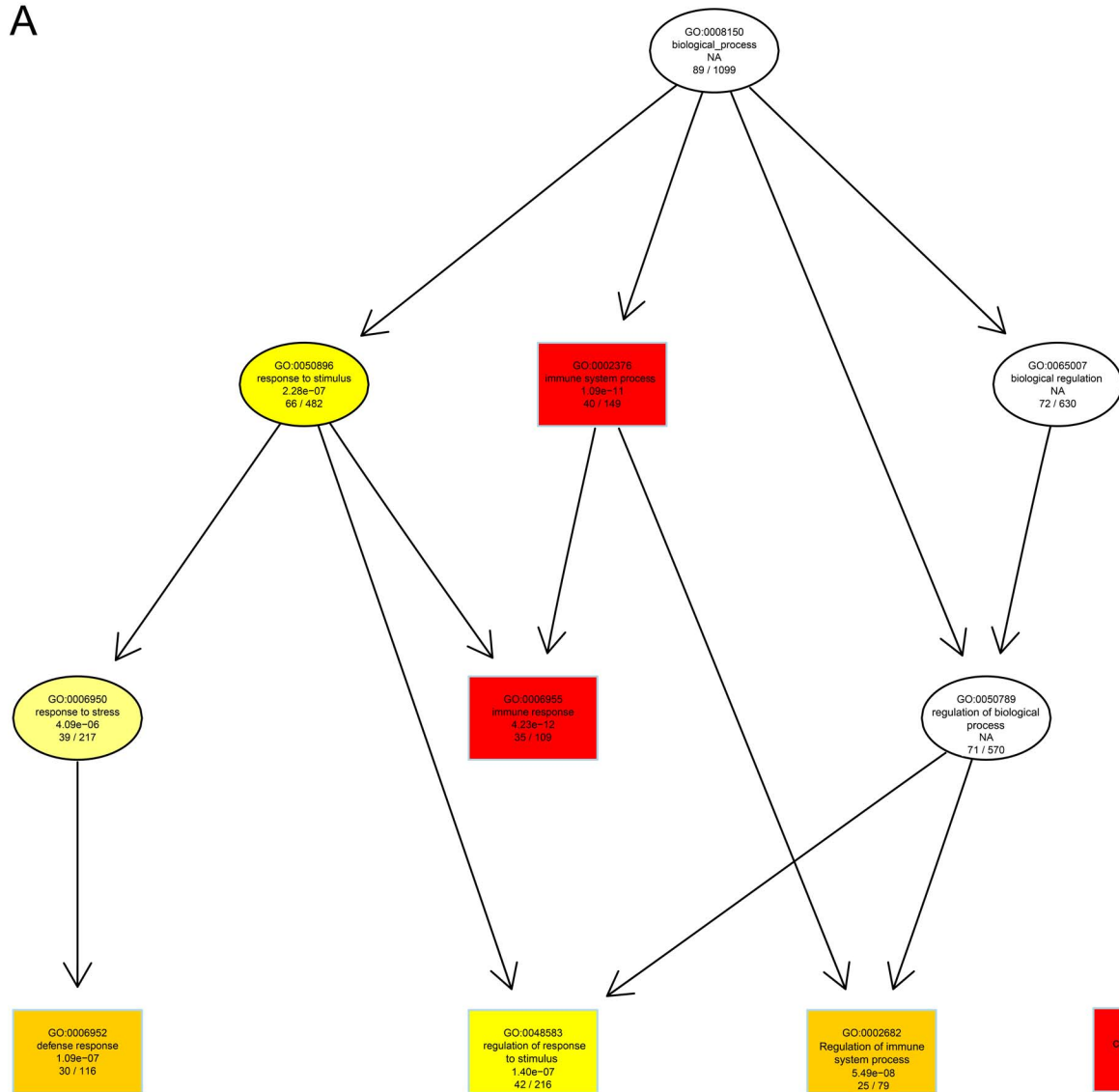

B

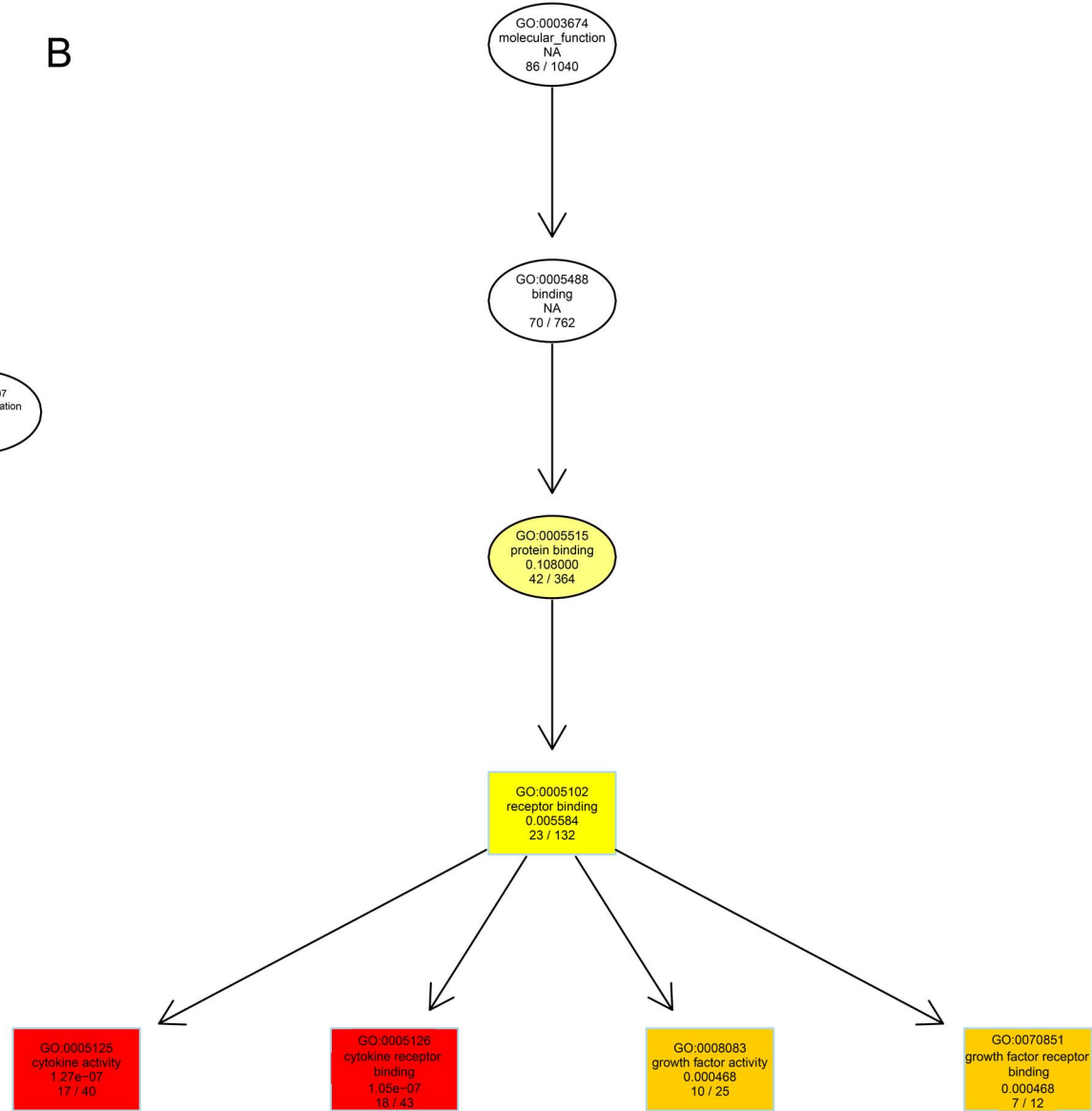

Supplement: Supplementary file 14 [file Data_Sheet_1.PDF]
