# Supplementary material for: RNA-Seq-Based Whole Transcriptome Analysis of IPEC-J2 Cells During Swine Acute Diarrhea Syndrome Coronavirus Infection
Source: Front Vet Sci. 2020 Aug 13;7:492. doi: 10.3389/fvets.2020.00492 (PMC7438718; doi:10.3389/fvets.2020.00492)
Supplement: Supplementary file 15 [file Data_Sheet_2.PDF]

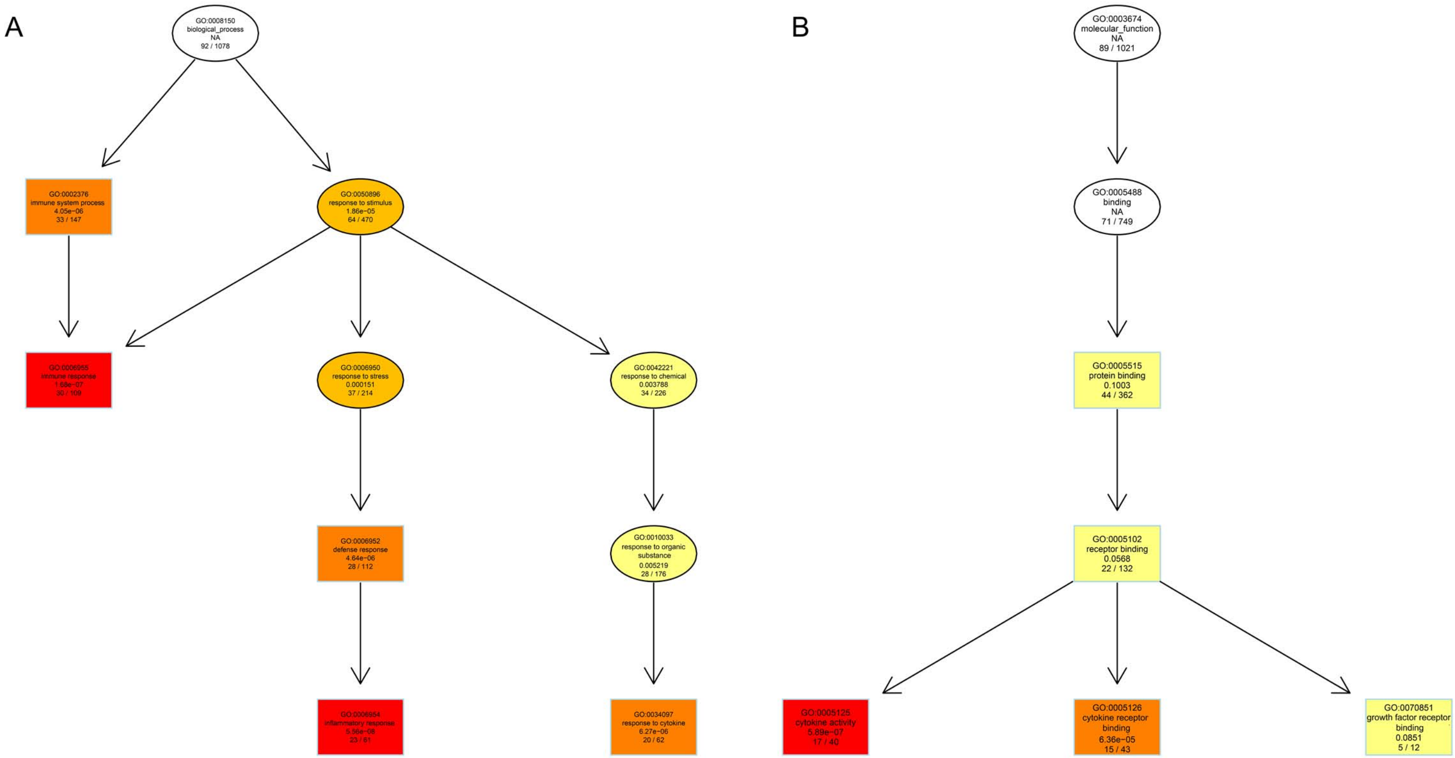

Figure S2. Significantly enriched GO biological process terms in the up- and down-regulated genes in SADS-CoV-infected IPEC-J2 cells at 48 hpi. Boxes and ellipses with colored background represent significantly enriched terms.
