# Supplementary material for: RNA-Seq-Based Whole Transcriptome Analysis of IPEC-J2 Cells During Swine Acute Diarrhea Syndrome Coronavirus Infection
Source: Front Vet Sci. 2020 Aug 13;7:492. doi: 10.3389/fvets.2020.00492 (PMC7438718; doi:10.3389/fvets.2020.00492)
Supplement: Supplementary file 16 [file Data_Sheet_3.docx]

Supplementary Material

## Supplementary Figures

**Supplementary Figure S1.** Significantly enriched GO biological process terms in the up- and down-regulated genes in SADS-CoV-infected IPEC-J2 cells at 24 hpi. Boxes and ellipses with colored background represent significantly enriched terms.

**Supplementary Figure S2.** Significantly enriched GO biological process terms in the up- and down-regulated genes in SADS-CoV-infected IPEC-J2 cells at 48 hpi. Boxes and ellipses with colored background represent significantly enriched terms.

## Supplementary Tables

**Supplementary Table S1.** The primers used for validation of the RNA-Seq data by real-time quantitative PCR.

**Supplementary Table S2.** Reads counts and quality of samples used in RNA-Seq.

**Supplementary Table S3.** Statistics of expressed genes in RNA-Seq in this study

**Supplementary Table S4.** Differentially expressed genes (log_2_|FC| ≥ 1 and *p*adj＜0.05) for comparison between infected and mock-infected samples at 6 hpi.

**Supplementary Table S5.** The directed acycline graph of the GO category in the biological process, indicating a significant enrichment in infected samples at 24 hpi.

**Supplementary Table S6.** The directed acycline graph of the GO category in the biological process, indicating a significant enrichment in infected samples at 48 hpi.

**Supplementary Table S7.** GO functional categorization of differentially expressed genes regulated in the samples of 6 hpi.

**Supplementary Table S8.** GO functional categorization of differentially expressed genes regulated in the samples of 24 hpi.

**Supplementary Table S9.** GO functional categorization of differentially expressed genes regulated in the samples of 48 hpi.

**Supplementary Table S10.** KEGG functional categorization of differentially expressed genes regulated in the samples of 6 hpi.

**Supplementary Table S11.** KEGG functional categorization of differentially expressed genes regulated in the samples of 24 hpi

**Supplementary Table S12.** KEGG functional categorization of differentially expressed genes regulated in the samples of 48 hpi

**Supplementary Table S13.** Verification of RNA-Seq results
